# The normative background of empirical-ethical research: first steps towards a transparent and reasoned approach in the selection of an ethical theory

**DOI:** 10.1186/s12910-015-0016-x

**Published:** 2015-04-04

**Authors:** Sabine Salloch, Sebastian Wäscher, Jochen Vollmann, Jan Schildmann

**Affiliations:** Institute for Medical Ethics and History of Medicine, Ruhr University Bochum, NRW Junior Research Group “Medical Ethics at the End of Life: Norm and Empiricism”, Malakowturm – Markstr. 258a, D-44799 Bochum, Germany

**Keywords:** Empirical-ethical research, Research methods, Ethical theory, Philosophy, Ethical interventions, Oncology

## Abstract

**Background:**

Empirical-ethical research constitutes a relatively new field which integrates socio-empirical research and normative analysis. As direct inferences from descriptive data to normative conclusions are problematic, an ethical framework is needed to determine the relevance of the empirical data for normative argument. While issues of normative-empirical collaboration and questions of empirical methodology have been widely discussed in the literature, the normative methodology of empirical-ethical research has seldom been addressed. Based on our own research experience, we discuss one aspect of this normative methodology, namely the selection of an ethical theory serving as a background for empirical-ethical research.

**Discussion:**

Whereas criteria for a good ethical theory in philosophical ethics are usually related to inherent aspects, such as the theory’s clarity or coherence, additional points have to be considered in the field of empirical-ethical research. Three of these additional criteria will be discussed in the article: (a) the adequacy of the ethical theory for the issue at stake, (b) the theory’s suitability for the purposes and design of the empirical-ethical research project, and (c) the interrelation between the ethical theory selected and the theoretical backgrounds of the socio-empirical research. Using the example of our own study on the development of interventions which support clinical decision-making in oncology, we will show how the selection of an ethical theory as a normative background for empirical-ethical research can proceed. We will also discuss the limitations of the procedures chosen in our project.

**Summary:**

The article stresses that a systematic and reasoned approach towards theory selection in empirical-ethical research should be given priority rather than an accidental or implicit way of choosing the normative framework for one’s own research. It furthermore shows that the overall design of an empirical-ethical study is a multi-faceted endeavor which has to balance between theoretical and pragmatic considerations.

## Background

Empirical-ethical research constitutes a relatively new field of enquiry which is characterized by the fact that socio-empirical research and ethical analysis are integrated for the treatment of concrete moral questions in modern medicine [1]. A broad variety of methodologies for empirical-ethical research has been suggested in recent years [2-4] and has been applied to concrete studies [5-7]. In this article, the argumentative structure upon which empirical-ethical research is based will be understood as “mixed judgments”, which contain both normative and descriptive or prognostic propositions ([8, p. 9]). Regarding the methodology of empirical-ethical research, all the different aspects of this argumentative structure should be considered: the justification and origin of the normative premises, the development of the empirical premises and the integration of both into an ethical judgment. However, not all of these parts are currently addressed in the literature on empirical-ethical research to the same extent.

The question of *normative-empirical interaction*, i.e. the interplay between empirical data and normative elements, has been extensively investigated in recent years [9-11]. Methodological questions related to the *empirical* research which forms part of empirical-ethical studies have also been debated to a considerable extent [12,13]. By contrast, the *normative* methodology of empirical-ethical research remains rather underexposed so far. Therefore, this article focuses on the normative side of empirical-ethical research methodology and aims to shed light on one particular aspect: based on our experience, we will make a suggestion of how to proceed in the selection of a normative background for a concrete empirical-ethical study. Our own ETHICO project (“Empirical-Ethical Interventions in Oncology”), which forms a multistep empirical-ethical study for the development of interventions supporting clinical decision-making, made us aware of key aspects which are relevant for the selection of a normative background.

In the first section of this article, we discuss several meta-theoretical preconditions which underlie the idea of theory selection in ethics. We then address the consequences which emanate from the pluralism of ethical theories for philosophical ethics and for the applied ethics domain. Subsequently, we will present three criteria which we encountered as relevant in our own empirical-ethical study: the adequacy of the ethical theory for the issue at stake, the suitability of the theory for the purposes and design of the empirical-ethical research project and the interrelation between the ethical theory selected, and the theoretical backgrounds of the socio-empirical research. These criteria will be illustrated by reference to the ETHICO project and the limitations of our theory selection will be discussed. The article closes with a short summary of the main points developed.

Our main aim in this article is to provide, based on our own experience, a first suggestion of how to develop a strategy for utilizing normative-ethical theories in empirical-ethical research. However, the topic of theory selection in empirical-ethical research touches on a number of fundamental problems in ethical theory and the philosophy of science, such as whether the plurality of normative-ethical theories can be reduced to one overarching approach [14] or whether a rational selection between scientific theories is possible at all [15,16]. These and other challenges can only be dealt with superficially in this article. We do not aim to provide answers to these highly controversial issues, but to stimulate and enhance the current research practice of empirical-ethical studies.

## Discussion

### Meta-theoretical considerations

The question of how to determine, justify and make the normative framework explicit for empirical-ethical research is a crucial one because direct inferences from descriptive data to normative conclusions are problematic for theoretical, methodological and pragmatic reasons [17]. Furthermore, depending on the normative background chosen, the impact of the empirical data for ethical judgment differs with regard to which type of data is needed and how it is processed within normative deliberation. There are different types of normative background which could be principally considered when conducting empirical-ethical research. Researchers can, for example, refer to a common morality or they can build on their private moral opinion. The potential of *philosophical-ethical theory* for concrete questions in medical ethics has been doubted for various reasons, such as a perceived lack of practical usefulness or problems arising from morally pluralistic societies [18,19]. However, the authors of this article appreciate the potential of philosophical-ethical theories to be utilized for empirical-ethical research. One reason for this position lies in the idea that philosophical-ethical theories (in contrast to theories which have a descriptive or explanatory character) do not primarily aim to fit the world as it is, but to guide human agency [20]. Due to this reverse “direction of fit” of philosophical-ethical theories compared to other types of theory, the question of the justification or well-foundedness of the theory is of particular importance in ethics. Ethical theories are usually based on elaborated accounts of normative justification. Building on such theories permits an external critical evaluation of the moral issues at stake. By contrast, only referring to the “lived morality” of stakeholders’ moral experiences and beliefs is fraught with the danger of perpetuating wrongful practices. In this respect, we see the benefit of utilizing philosophical-ethical theories for empirical-ethical research.

This article should generally be understood as a plea for a more transparent and reasoned approach in selecting an ethical-theoretical background for empirical-ethical research. Consequently, we will start from the assumption that there is a plurality of coexisting normative-ethical theories which could be applied to a concrete issue [21]. In contrast to philosophy of science, where skepticism towards a universal theory of justification has been widespread since the beginning of the 20^th^ century, the idea of a comprehensive theory is still vivid in ethical theory ([22], p. 312 f.). However, a generally accepted overarching ethical meta-theory, integrating all accounts without losing their specific perspectives, is not available. Following Julian Nida-Rümelin’s coherentist approach, the plurality of normative-ethical accounts mirrors the variety within our actual moral thinking ([22]; p. 314 f.). In our everyday moral judgments, we operate with a diversity of moral concepts and criteria, such as rights, duties and principles. Theory selection in empirical-ethical research, along these lines, can be understood as the question of what aspect of moral thinking should have priority in the current discussion. While different theoretical accounts could be applied similarly to a certain subject there are also reasons, why one theory might be a better fit than another. Our article tries to elucidate some of these reasons and to make a suggestion of how to proceed explicitly and deliberately in theory selection.

The issue of theory selection is not a matter of rational argument alone. In research practice, personal and biographical factors and pragmatic considerations of acceptance in the scientific community have a strong influence on researchers’ decisions regarding which ethical theory to choose. Reflection on the researchers’ own socio-cultural embeddedness is, thus, crucial for dealing with conflicts of interest and biases in bioethical research [23]. In the context of this article, we would like to stress the need to develop a critical stance towards one’s own ethical-theoretical commitments based on systematic criteria for theory selection. Along these lines, we will start by discussing the consequences which arise from the plurality of normative-ethical theories for philosophical ethics and applied ethics.

### Dealing with the pluralism of ethical theories

In philosophical ethics, the plurality of normative theories does not usually lead to major practical problems. By contrast, within the ethical-theoretical sphere, the diversity of accounts about normative justification often serves as a reference point for fruitful discussions about ethical concepts and the general nature of morality. There is also an intensive reflection in philosophy about the interrelation between different types of theory, such as contractualist and consequentialist accounts. In general, a large number of current discussions in philosophical ethics are (next to meta-ethical topics) dedicated to issues of discussing, modifying and combining divergent accounts of normative justification.

This situation of (more or less) harmonic coexistence changes when we leave the theoretical realm and enter the field of applied ethics. The relationship between the emerging branch of empirical-ethical research and the more traditional idea of “applied ethics” is ambiguous. In this article, the term “applied ethics” is used in a rather broad sense, and is not restricted to one particular methodology or to so-called “top down” -approaches ([24], p. 321 ff.). Empirical-ethical research will, therefore, be regarded as one option to follow working in applied ethics. Applied ethics (including empirical-ethical research) is supposed to deal with concrete ethical problems. If normative solutions are provided, this often has far-reaching consequences for society and the future of individuals. Using different ethical theories as starting points can lead to divergent answers regarding concrete ethical problems [24,25]. A main difference can be observed between consequentialist theories and theories arguing on a deontological basis, for example, in referring to human dignity. These approaches often result in divergent normative evaluations, for example with regard to the question of how much protection must be given to early forms of human life. Hence, it often makes a great difference in the sphere of applied ethics whether a certain problem is treated on one ethical-theoretical background or the other. Or, as Konrad Ott puts it: “If you are unlucky, you will catch an adherent of Singer, Tooley or colleagues when you are a disabled infant, or as an asylum seeker, somebody who defends a mixture of ‘hard’ communitarianism and evolutionary ethics” ([26], p. 73; own translation).

The selection of an ethical theory underlying one’s own research is, thus, a crucial factor which influences the outcome of applied ethics, including empirical-ethical research. Unfortunately, the topic is rarely addressed in textbooks or introductory seminars on applied ethics. Philosophical-ethical theories are presented and discussed much more often without much explanation regarding their inherent relatedness or the ways of selecting one for the treatment of a concrete ethical issue [27-29]. It is also rather uncommon for authors writing in applied ethics to declare explicitly why they feel themselves committed to a specific ethical theory ([30], p. 62). In some instances, this may be an issue of the author’s individual preferences or a byproduct of their academic socialization. However, in light of the practical relevance of applied ethics work, the selection of a normative background is far from being of mere scholarly interest. Instead, it is closely linked to the researcher’s commitment to diligence and transparency and, thus, can be regarded as a core aspect of the ethics of carrying out ethics research.

### Criteria for theory selection: theoretical considerations and their application in the ETHICO project

How, then, can the selection of a normative background proceed with regard to empirical-ethical research? In the following, three criteria for theory selection which we encountered as being relevant in our own project will be discussed. The ETHICO project aims at the development of interventions which support clinical decision-making on an empirical-ethical basis. It has a double-tracked structure encompassing qualitative empirical research as well as normative analysis. The interrelation between descriptive and normative aspects and theories applied is of central interest within the project. The ETHICO project has a model character and takes place in the oncology department of one German hospital [31]. It consists of six stages which range from the identification of ethical problems up to the development, implementation and evaluation of an intervention to support the clinical decision-making. The general structure of the project can be seen below:

#### The ETHICO project (“Empirical-ethical interventions in oncology”)

- **Main Features:**
  - → development of interventions to support clinical decision-making in oncology
  - → empirical-ethical methodology
- **Stages:**
  1. “Preparation“
    development of a framework for the empirical-ethical intervention project
  2. “Exploration*“*
    qualitative research to identify ethical challenges in the context of an oncologic department
  3. “Deliberation“
    development of an empirically and normatively founded solution for the ethical problems identified
  4. “Development“
    development of empirical-ethical interventions to support the decision making
  5. “Intervention“
    implementation of the interventions (communication guidance for the ward team, leaflet with questions for the patients)
  6. “Evaluation“
    evaluation of the intervention and the overall study concept.

The requirements for the selection of a normative background in the ETHICO project differ from both philosophical ethics and “general” applied ethics. In *philosophical ethics*, normative theories are typically understood as systematic accounts regarding the question of what constitutes morally right or wrong actions, or – in an evaluative sense – good or bad human conduct ([32], p. 1). Criteria for the good quality of an ethical theory are mainly related to aspects which are inherent to the theory itself, such as clarity, coherence and simplicity ([33], p. 352 ff.). These criteria are not only valid for ethical theory, but are also well-known from the philosophy of science regarding other fields of scientific enquiry. A main focus in philosophical ethics, thus, lies on the well-foundedness and acceptability of a normative-ethical account, while only rarely other more practice-related requirements towards an ethical theory are discussed.

When we enter the field of *applied ethics*, additional criteria become relevant regarding the question of what constitutes a “good ethical theory.” Applied ethics, as a practice-related discipline, is supposed to deliver answers and support with regard to concrete and often urgent ethical issues, such as challenges arising from new technologies or societal developments. Applied ethics is, thus, expected to provide a normative orientation which is helpful in practice. To develop such an action-guiding potential, the normative theory applied, for example, needs to be compatible with the stakeholders’ actual moral thinking and behavior ([24], p. 325). Therefore, not only the theory’s acceptability (in the sense of its well-foundedness), but also its factual acceptance by the stakeholders has to be considered. Hence, ethical theories in applied ethics – more than in the “pure” philosophical realm – gain the character of “problem-serving tools”. This leads to additional, more pragmatic requirements becoming relevant compared to the use of the theories in the philosophical ethics field [34].

In the specific interdisciplinary field of *empirical-ethical research*, of which the ETHICO project forms part, even more facets must be considered for the selection of a normative background. Socio-empirical research in ethics serves a variety of different aims, ranging from the mere description of ethically relevant attitudes, through normative evaluations of the respective practices, to intervening in practice to modify people’s behavior [9,35]. Furthermore, empirical-ethical research forms a particularly intricate research field, as the demands of empirical research methodology have to be integrated with criteria for a good normative-ethical analysis [36]. In the following, we will discuss three relevant requirements which we encountered in our own empirical-ethical study. The aspects do not provide an exhaustive list of all relevant considerations; other aspects are also important, such as the researchers’ competence to deal with certain theoretical concepts or the acceptance of the theory’s results by the stakeholders involved. The three points discussed can, thus, be seen as main examples for what has to be considered when selecting the normative background for an empirical-ethical research project. All three aspects will firstly be first presented in a more abstract manner. Subsequently, the concrete outcome of theory selection in the ETHICO project will be briefly sketched.

#### The adequacy of the ethical theory for the issue at stake has to be considered in the selection of a theoretical background

An ethical theory which is selected to underlie an empirical-ethical research project has to fit the study’s thematic subject. Subject here means the practical problems and the empirical context to which the study is directed. This idea of a fit between an ethical theory and its subject needs further explanation: Most philosophical-ethical theories are – by their own self-understanding – not restricted to a specific field of practice, but are supposed to guide human action in general ([26], p. 73). However, normative concepts and principles contain descriptive elements [37] in referring to aspects of human life, such as happiness, preferences, moral experience, and much more. Hence, empirical characteristics form part of normative premises which are integrated into ethical judgment. It can, therefore, be argued that an ethical theory fits a certain subject if it is in line with salient features of the issue which is under discussion. Ethical theories can also fail in this respect, by not matching particular problems. Utilitarianism, for example, has its focus on particular aspects of the social reality (such as benefit and harm), but does not capture other aspects which are of equal importance to understand the moral phenomenon under discussion. A utilitarian account, therefore, might not adequately address ethical questions about caring relations at the end of life.

A second line of argument which is important for the fit between an ethical theory and the issue at stake is the more pragmatic consideration of an alignment between the ethical-theoretical background and the stakeholders’ actual moral deliberation and behavior, which form part of the moral phenomenon observed. If the ethical theory applied does not fit the stakeholders’ actual moral thinking, ethical interventions might not be successful ([24], p. 321). Therefore, the ethical theory selected also has to be in line with the lived normativity already embedded in practice.

It is also important to take into consideration that theories which, at first glance, do not seem to fit a certain subject very well, bear the potential of shedding a new and fresh light on the issue at stake, especially if they highlight aspects which might have been overlooked without that specific theoretical perspective. Using a virtue ethics framework for questions of distributive justice, for example, might not be the most obvious option, but helps to gather a new and broader understanding of the issue. The capability approach, for instance, rests on the idea of human thriving and, therefore, goes beyond “traditional” approaches to distributive justice which focus on personal utility, negative freedoms or comparisons of resource holdings [38].

Considering the relationship between an ethical theory and the specific research topic is relevant for applied ethics in general. However, it occurs in a specific form with respect to empirical-ethical research. Empirical-ethical research bears the characteristic that many features of the issue at stake are not well-known prior to the empirical research, because a better knowledge about the empirical characteristics is one of the expected outcomes. The fit of a theoretical background, therefore, often cannot be fully evaluated *ex ante* in empirical-ethical research. How then can the matching between the study’s subject and the ethical theory proceed in empirical-ethical studies? Different strategies could be principally applied here. A first option would be to determine the ethical-theoretical background before starting on the empirical research. As – depending on the theory chosen – different types of data are needed (e.g. a preference utilitarian needs different empirical data for the ethical analysis compared to a virtue theorist [17]), the choice of a specific theory has a strong influence on the selection and processing of empirically gathered data. This would lead to a preponderance of ethical theory which might contradict the methodological requirements and aims of social science research – especially in the case of qualitative social research. A second strategy would, thus, be to conduct the empirical research first and then designate an ethical theory for normative evaluation. However, going along this road would miss the potential of ethical theories for gaining a more comprehensive understanding of an empirical issue. Therefore, the third (and preferable) strategy is to stay rather undetermined in terms of ethical theory before the beginning of data gathering. If the field is then approached empirically, the researcher has the opportunity to select a theory which is best suited to capture the relevant features of the ethical problem. A constant interchange between the normative theory selected and the empirical data ensures that both sides are respected as playing an important role in the empirical-ethical research process ([4], p. 473 f.). The reflection has a cyclical character: the empirical phenomenon is understood against the background of the ethical theory and, on the other hand, helps to provide a deeper understanding of the semantic implications of the normative concepts and principles applied.

It was important for the ETHICO project that the normative background could be linked with the core features of oncologic practice, especially with empirical aspects of decision-making in advanced cancer. However, the researchers did not have full knowledge about the clinical practice in the department before conducting the empirical research. A spectrum of possibly suitable ethical theories was, therefore, prepared before the beginning of the empirical research based on a literature review which focused on normative theories which had been applied to similar issues previously. Our empirical study then consisted of a triangulation of qualitative methods (interviews, non-participant observation and focus groups). During our empirical research, we were aware of the spectrum of ethical theories which could be applied for a normative analysis and we correlated this with our (preliminary) empirical results. The final selection of a theoretical background was not made until the qualitative research had progressed to a stage which allowed us to gather a first understanding of the most important empirical characteristics of the social phenomenon observed. The ethical theory subsequently selected had an influence on the further qualitative data gathering, while, at the same time, the empirical data collected were used to specify and adjust the ethical theory with respect to the particular context. Hence, in a circular process, the selection of an ethical framework was considered during the empirical research, while, simultaneously, the theory selection was influenced by our preliminary empirical results.

As a result of preliminary data collection and analysis, we identified the issue of respect for the patient’s will as being important in the different settings of decision-making (tumor conference, ward round, outpatient clinic). This finding could be related to concepts of patient autonomy from the medical-ethical literature. It also became obvious that the way in which patient autonomy is exercised in practice is very much dependent on structures of decision-making [31]. We, therefore, decided to select an ethical theory which did not conceptualize autonomy in a liberalistic way but stressed the importance of institutional structures which promote patient autonomy [39].

#### The selection of a normative background has to account for the purposes and design of the empirical-ethical research project

Empirical research in medical ethics can contribute to normative arguments in a variety of ways. Examples are the identification of morally relevant problems, the provision of facts important for normative arguments, the description of the actual conduct of a group of stakeholders [9], the analysis of moral concepts, the construction of a normative standpoint, or the implementation of an intervention to enhance the moral quality of a practice ([30], p. 45 f.). Therefore, the purposes and design of concrete empirical-ethical studies can vary to a considerable extent. One ethical theory might be more adequate than others, depending on the exact aims of the empirical-ethical research project. The idea of the “application” of an ethical theory to a concrete issue should be understood as having a three-sided relationship: something is applied to something to some end ([26], p. 58 f.). “Application,” thus, always carries a teleological momentum, as it is inherently related to a purpose to which the application is supposed to serve. The selection of a normative background, therefore, should take into account the purposes of the concrete research project.

There are some approaches in the spectrum of philosophical-ethical theories which seem to be quite well-fitted to develop clear normative guidance. Other approaches are better suited to providing rich and comprehensive descriptions of moral phenomena. This latter type can be designated as “normatively weak” approaches [40]. This does not mean that these theories are less elaborate. Instead, they are not primarily directed towards normative evaluation, but serve better for other purposes. One example of such a “normatively weak” approach would be narrative ethics [41,42]. Narrative ethics uses the medium of stories to better understand phenomena such as sickness, dependency or disability, which are of great importance for medical ethics. Therefore, it serves different aims than ethical theories which have been developed to serve concrete practical (e.g. political) purposes and, consequently, carry a stronger action-guiding character. Classical utilitarianism, as presented by John Stuart Mill, for example, is based very much on the author’s political ideas and targets concrete suggestions for an improvement of practice. Utilitarianism is, thus, more than narrative ethics directed towards normative evaluation. While both types of theory can contribute principally to an improvement of practice, their different aims and character should be considered in the selection of a normative background. If an empirical-ethical research project is, for example, supposed to contribute to a clear-cut normative evaluation, it would be advisable to select an ethical theory which allows for concrete action-guiding suggestions. If the project aims more at a fuller understanding of a moral phenomenon, a “normatively weak” approach, such as narrative ethics, might be the better choice.

In the ETHICO project, we had to face the challenge that the ethical theory selected was supposed to serve a variety of aims during the different stages of the research (see section The ETHICO project (“Empirical-ethical interventions in oncology”)). In the “deliberation” stage, for example, the ethical-theoretical framework was needed to guide the normative deliberation aiming at an ethically justified assessment of the moral problems which had been identified in the earlier project stages. Therefore, the ethical theory had to indicate what would be an appropriate solution to the moral deficits observed. Another example would be the “evaluation” stage, where a normative background was needed to determine whether the ethical quality of the practice had improved subsequent to the empirical-ethical intervention. The development of evaluation criteria is of great importance here. While there are some ethical theories (such as utilitarianism) which may even allow for a quantitative measurement, other theories (e.g. Kantian accounts) may not provide good opportunities for an empirical evaluation of the ethical quality of a practice.

The diverging aims of the different project stages finally led us to the conclusion that it would not be advisable to stick to one and the same ethical theory throughout the project. Instead, we drew on different normative backgrounds (partly in combination), depending on the purposes of each stage in the ETHICO project. During its first stages, which aimed at the identification and characterization of ethical problems, we stayed rather open and considered different theories which are suitable to deliver an empirically rich understanding of the moral phenomenon observed (such as narrative ethics or virtue ethics accounts). In stage three, “deliberation”, which aimed at the development of an ethically justified solution, we referred to O’Neill’s account on principled autonomy [39], which also had an influence on the intervention, which was then designed to establish trustworthy structures in the respective oncological department. In the final “evaluation” stage, we included, in addition to O’Neill’s account, Alan Gewirth’s theory, which considers basic structures of human agency [43]. We then tried to assess how far patients were enabled to exercise their right to self-determination in hospital,and whether they are hindered by factors, such as misinformation or a symptom burden, which do not allow them to exercise their autonomy.

The combination of diverse ethical theories and, thus, different approaches to ethical justification (e.g. virtues ethics and Kantian accounts) became necessary due to the comprehensive structure of the overall project, which is an empirical-ethical intervention study and, therefore, combines different aims. In addition, this approach mirrors our meta-theoretical view that the plurality of normative-ethical accounts corresponds to the variety within actual moral thinking. It was, therefore, necessary to draw on more than one ethical theory to deal adequately with the multifaceted phenomenon of decision-making in advanced cancer from a normative perspective.

#### The interrelation between the ethical theory selected and theoretical backgrounds of the socio-empirical research should be reflected

Empirical-ethical research includes socio-empirical inquiry as well as normative analysis. Hence, methodological requirements and theoretical backgrounds on “both sides” and their interrelationship should be taken into consideration throughout a project [36]. The choice of an ethical theory, therefore, has to consider the interrelation between the ethical theory selected and the theoretical backgrounds of the empirical research which forms part of the study. Empirical research methodologies carry certain normative assumptions which can be either in line or in tension with central ethical concepts. It has been stressed, for example, that quantitative surveys constitute society and subjects in a way in which the survey method can succeed [44]. This may contradict ethical notions, such as civic liberty or the person’s right to self-determination.

Besides this implicit normativity, socio-empirical research is laden with theoretical presuppositions about the social reality observed. Social theories make assumptions about what counts as a social phenomenon and which concepts should be regarded as core items in this context (e.g. agency, interaction or communication) ([45], p. 237). Such socio-theoretical presuppositions, which underlie empirical data gathering and analysis, and their relationship to the ethical theory selected should be respected when planning and conducting an empirical-ethical study [45]. There are some social theories which share the central premises of main philosophical-ethical theories, while others rest on assumptions which cannot be linked to ethics so easily. One “linking element” in the first respect would be the assumption that there are rational actors ([45], p. 239). This idea underlies, for instance, Grounded Theory methodology [46], which is based on the concept of interactiondeveloped by George Herbert Mead [47]. Regarding the idea of actors being the main concept, Grounded Theory stands in a “harmonic relationship” with most ethical theories which similarly focus on action ([45], p. 239). In other respects, a tension can be noticed when Grounded Theory is applied in the context of ethics. The understanding of reality, for example, as a social interaction with others and shared interpretative process is in contrast with those ethical theories which have a rather individualistic account of human agency.

The tension between ethics theory and social theory also becomes obvious when other socio-theoretical backgrounds are taken for empirical-ethical studies. One example in this respect would be systems theory, developed by Niklas Luhmann [48,49]. In systems theory, actors are not the main concept, therefore, it cannot be as easily aligned with ethical theory. We do not want to suggest that social theory and ethical theory should be congruent in all relevant features, however, researchers should be aware of whether the ethical theory they select harmonizes or stands in a strained relationship with the socio-theoretical presuppositions on which they draw. When this is not respected, there is a danger of missing ethically problematic issues if they are not in the focus of the social theory which underlies the empirical research.

We decided on a qualitative research methodology in the ETHICO project. We used an observational research method which is based on Symbolic Interactionism [50] in the “exploration” stage to investigate the clinical practice with regard to the question, which ethical problems are relevant and how can they be further characterized [31]. We tried to understand and reconstruct the meanings which certain objects and actions had for the research participants. An underlying presupposition of Symbolic Interactionism is the existence of intentional actors who stand in relationship with other actors and choose means to achieve their aims. This interaction is based on processes of mutual interpretation. The socio-theoretical model of intentional actors can be linked to many approaches from the spectrum of philosophical-ethical theories, which equally start from the assumption of purposeful human agency.

Therefore, the socio-theoretical framework selected gave us ample scope for the choice of an appropriate ethical theory in the ETHICO project. All ethical theories selected rest on the assumption of actors who make their choices on the basis of preferences and information. In addition, O’Neill’s non-individualistic account of autonomy stresses the importance of (social) structures and human interaction and can, therefore, serve as a linking element between the social-theoretical reconstruction and the ethical reflection on social practices in oncological decision-making.

### Limitations

We generally tried to apply a transparent and reasoned approach regarding the choice of a normative background in the ETHICO project. However, the issue of theory selection appeared to be highly complex and led to a need for compromises, especially with regard to the tension between the theoretical well-foundedness of normative-ethical accounts and their applicability in practice. As displayed above, there are criteria inherent to an ethical theory which are important for judging its quality. If an ethical theory is insufficient with respect to clarity and coherence, it should not be applied in empirical-ethical research – even if it fits the designated subject very well, is in line with the overall aims of the project and matches the project’s socio-empirical research methodology very nicely. In addition to these three aspects which pertain to the appropriateness of a theory within a specific project, normative theories fulfil a critical function. When there is no theoretical justification apart from the suitability for the research, this external critical evaluation is not executed. A theoretical justification independent of the concrete context of application is crucial for empirical-ethical research to develop a critical stance towards the social practice observed. However, even the best-founded ethical theory cannot be successfully applied when any link between the theoretical notions and the empirical reality is missing. Researchers into the practice of empirical-ethical study should be aware of this ambivalence and the problems which arise from a preponderance of either the theory’s well-foundedness or its suitability within the concrete project.

A second important result emerging from our research is the need to apply diverging ethical theories within the different stages of the ETHICO project. This necessity mainly arises from the varying purposes we had during the research project: from the designation of an ethical problem up to the designing, implementation and evaluation of an intervention. While one ethical theory serves better for a deeper understanding of the relevant empirical phenomena, other normative backgrounds are better suited to measure the ethical quality of a social practice. This finding suggests that ethical theoriesin empirical-ethical research ethical theories cannot often be used in their “pure” form but have to be further modified to meet the requirements of the topic chosen and the aims of the respective research. A syncretic combination of different theories, which might be regarded as problematic from a philosophical perspective alone, can be justified in the context of an empirical-ethical research project. However, arbitrariness in theory selection should be avoided by explaining the reasons why certain approaches have been chosen, modified and combined for a concrete project.

## Summary

In this paper, we aimed to stress that the specific set-up of empirical-ethical research necessitates a comprehensive reflection with regard to the selection of an ethical background theory. In contrast to traditional research in philosophical ethics, additional issues should be considered here which pertain to pragmatic aspects of conducting the research, as well as to more theoretical facets, such as the interrelation between the ethical-theoretical and the socio-theoretical background of the respective study.

It also became obvious that the requirements which are imposed on an ethical theory in the empirical-ethical field are rather high. Based on our practical experience in the ETHICO project, we have learned that there is probably no single ethical theory which fully accomplishes all the criteria relevant for theory selection. As a *jack of all trades* device consistent with all aspects discussed above will not be found in the spectrum of ethical theories, researchers should consider a modification or combination of different accounts. The overall design of an empirical-ethical study is a multi-faceted endeavor which has to balance between more theoretical and rather pragmatic considerations.

In summary, this paper aimed to show that a systematic and reasoned approach towards theory selection in empirical-ethical research should be given priority compared to an accidental or implicit way of choosing the normative framework for one’s own research (or to only referring to the researcher’s personal moral stances). The criteria discussed above may, therefore, serve as relevant points to consider when it comes to the matter of theory selection in the planning and conducting of an empirical-ethical research project.
